# Facile and Sensitive Detection of Nitrogen-Containing Organic Bases with Near Infrared C-Dots Derived Assays

**DOI:** 10.3390/nano11102607

**Published:** 2021-10-03

**Authors:** Chunyu Ji, Yiqun Zhou, Wenquan Shi, Jiajia Wu, Qiurui Han, Tianshu Zhao, Roger M. Leblanc, Zhili Peng

**Affiliations:** 1 National Center for International Research on Photoelectric and Energy Materials, School of Materials and Energy, Yunnan University, Kunming 650091, China; jichunyu@mail.ynu.edu.cn (C.J.); shiwenquan1010@163.com (W.S.); 12019101477@mail.ynu.edu.cn (J.W.); hqr3155064793@163.com (Q.H.); zhaotianshu@mail.ynu.edu.cn (T.Z.); 2 Department of Chemistry, University of Miami, 1301 Memorial Drive, Coral Gables, FL 33146, USA; yxz431@miami.edu (Y.Z.); rml@miami.edu (R.M.L.)

**Keywords:** carbon dots, nitrogen-containing organic bases, colorimetric sensor, spectral sensor, nitrogen fertilizer

## Abstract

In this article, we have designed both colorimetric (including solution and test paper type) and spectral sensors (including UV-vis and PL type) for the quick and sensitive detection of general nitrogen-containing organic bases (NCOBs); the limit of detection could reach as low as 0.50 nM. NCOBs included 11 examples, covering aliphatic and aromatic amines, five- and six-membered heterocyclics, fused-ring heterocyclics, amino acids, and antibiotics. Furthermore, the assays demonstrated high reliability in sensing NCOBs and excellent ability to distinguish NCOBs from oxygen and sulfur containing organics. The assays developed could find important applications for the detection of NCOBs in the fields of biomedicine, chemistry, and agriculture.

## 1. Introduction

Nitrogen-containing organic bases (NCOBs)—such as aliphatic amines (e.g., butylamine, spermine), aromatic amines (e.g., aniline), five-membered heterocyclics (e.g., pyrrole, imidazole), six-membered heterocyclics (e.g., pyridine), fused-ring heterocyclics (e.g., indole, quinoline, purine), amino acids (e.g., lysine), and antibiotics (e.g., tetracycline)—are widely used in biomedicine, chemical, agricultural, and other fields [1,2,3]. These NCOBs are very important by playing essential roles in all areas of human life: they are not only important fine chemical raw materials, but also have a variety of biological activities. For instance, as a natural polyamine, spermine plays an important role in cell growth and proliferation [4]. It can also act as an antioxidant and a metabolic regulator, and is an important indicator of malignant tumors. Tetracycline and its derivatives are widely used antibiotics in medicine, which can inhibit the synthesis of bacterial proteins to achieve antibacterial effects [5]. Meantime, NCOBs could also cause significant problems—such as environment pollution, adverse biological reactions, and food contamination—if not properly monitored (i.e., released into environment in high concentrations; accumulated in body in high amount, etc.). Thus, it is important to have simple and reliable sensing assays for the detection and determination of these NCOBs.

Unfortunately, limited by the sensing platforms, the sensitive detection of NCOBs is still very hard to achieve nowadays. Currently, there are only sporadic reports for the detection of NCOBs, in which sensing assays could only be applied to specific NCOBs and generally with low sensitivity [6,7,8,9]. A simple assay for the quick and sensitive detection of the general NCOBs is still missing from literature. On the other hand, C-dots are a new member of zero-dimensional carbon-based nanomaterials, with diameters less than 10 nm, and are known for their unique photoluminescence (PL) properties [7]. Compared with traditional semiconductor-based quantum dots (QDs) and organic dyes, C-dots have significant advantages in sensing due to their excellent PL, high stability, low toxicity, environmental friendliness, as well as easy and economical access [8,9]. As such, C-dots have been widely used for the sensing of metal ions, small molecules, and biomolecules [10,11,12].

**Figure 1:**
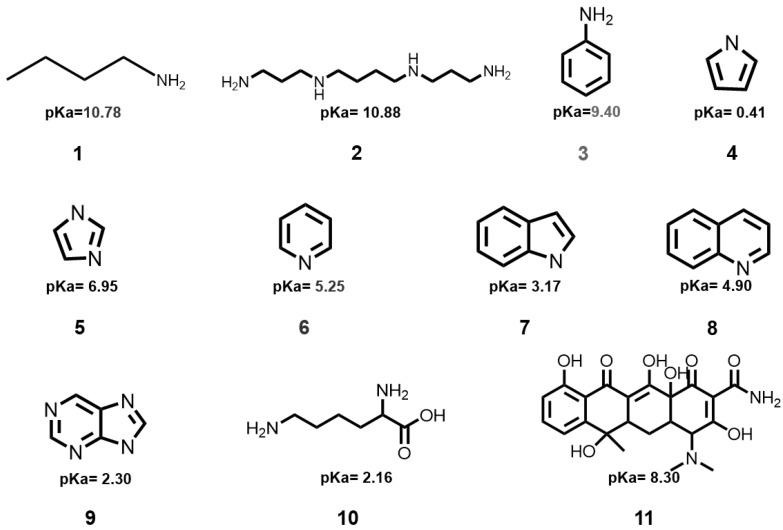
Chemical structures of NCOBs discussed in this article as well as the pKa of their conjugated acids (CA), the pKa values were obtained from reference [13].

In this context, for the first time, we report a C-dots-derived assay for the quick and sensitive detection and determination of general NCOBs. In this assay, the as-prepared C-dots without any additional modifications could be applied for the sensing of NCOBs. Most importantly, the analytes of this assay are not limited to a specific target; it could cover a broad scope of NCOBs (Figure 1), including aliphatic amines such as butylamine (**1**), spermine (**2**), aromatic amines such as aniline (**3**), five-membered heterocyclics such as pyrrole (**4**), imidazole (**5**), six-membered heterocyclics such as pyridine (**6**), fused-ring heterocyclics such as indole (**7**), quinoline (**8**), and purine (**9**), amino acids such as lysine (**10**), and antibiotics such as tetracycline (**11**). With this assay, both colorimetric (including solution, as well as test paper types), and spectral sensors (including UV-vis absorption and PL types) have been developed. The colorimetric sensors provide simple solutions for the quick and qualitative detection of general NCOBs; while the spectral sensors provide quick access for the quantitative detection of NCOBs with high accuracy and sensitivity, limit of detection (LODs) of these NCOBs could reach as low as 0.50 nM.

## 2. Materials and Methods

### 2.1. Reagents and Apparatus

Methanol (99.5%), hydrochloric acid (HCl 36–38%) and sulfuric acid (H_2_SO_4_ 95–98%) were purchased from Chengdu Chron Chemicals Company (Chengdu, China). o-phenylenediamine (o-PDA) (98%), tert-butyl hydroperoxide (TBHP) (70%), 3-chloroperbenzoic acid (85%), butylamine (99%), aniline (99.5%), pyridine (99%), pyrrole (99.9%), indole (99%), quinoline (99%), imidazole (98%), spermidine (99%), lysine (98.5%), tetracycline (98%), purine (98%), and N, N-Diisopropylethylamine (98%) and 8-Diazabicyclo [5.4.0] undec-7-ene (99%) were purchased from Energy Chemical Company (Shanghai, China). All the reagents were used as received without further purification, unless otherwise noted. The deionized water used in all experiments was made from a Master Touch-S laboratory ultrapure water machine (Master Touch, Shanghai, China).

### 2.2. Synthesis of C-Dots

We used o-PDA as the carbon source to synthesize C-dots. Specifically, a mixture of o-PDA (0.25 g), TBHP (0.2 mL), HCl (2 mL), and deionized water (20 mL) was heated in a microwave synthesizer (Discover SP of American CEM, Matthews, NC, USA) at 150 °C for 20 min. The reaction solution is then transferred to a 70 mL autoclave and heated at 200 °C for 12 h. After the reactor was cooled to room temperature, the solution was filtered and the precipitate was washed with 500 mL of deionized water. Then precipitate was vacuum dried to result in a black solid powder.

### 2.3. Characterizations of C-Dots

The absorption spectrum of C-dots (5 μg/mL) was detected by an ultraviolet-visible spectrophotometer (UV-vis, UV-2600, Shimadzu, Japan). Put 3 mL of methanol into two 1 cm × 1 cm × 3 cm cuvettes, and then put the cuvettes into the base of UV-vis to test the solvent baseline. The wavelength range is 195–1100 nm, the scanning speed is medium speed, the sampling interval is 1 nm, and the sampling times are repeated twice. Put 3 mL C-dots (5 μg/mL) into a 1 cm × 1 cm × 3 cm cuvette, and then put them into a UV-vis base to test the absorption spectrum.

The fluorescence spectrum of C-dots (5 μg/mL) was tested by a fluorescence spectrometer (FL, F97 Pro, Shanghai Prism Technology Co., Ltd., Shanghai, China). Put 3 mL of C-dots solution into a 1 cm × 1 cm × 3 cm cuvette and put it into the FL base to test the fluorescence spectrum. The scanning mode is three-dimensional wavelength scanning. The excitation wavelength is 200–900 nm, and the excitation width is 10 nm. The emission wavelength is 200–900 nm, and the emission width is 10 nm. The scanning speed is 1000 nm/min, and the scanning interval is 1 nm. The gain is 650 V.

The infrared absorption spectrum of C-dots was tested by Fourier infrared spectrometer (FTIR, Middle Age Walker, Thermo, Waltham, KS, USA). Put 200 mg potassium bromide into an agate mortar and fully dry it at 150 °C (30 mins), add 1.5 mg C-dots and grind it thoroughly. Then dry it at 100 °C for 5 mins, and then continues to grind for 30 s. Put the grounded powder into a mold and use a 10 t hydraulic press to press tablets. Put the pressed sample into the FTIR to test the infrared absorption spectrum.

The surface charge at C-dots was tested by a Zeta potential analyzer (Zeta potential, sample cell DTS1060, Malvern, Mulvane, UK). The particle size of C-dots was measured by transmission electron microscope (TEM, JEM-2100, Tokyo, Japan). The acceleration voltage is 200 kV, and the magnification is 800,000 times. The elemental composition of C-dots was tested by multifunctional X-ray photoelectron spectroscopy (XPS, K-Alpha, Thermo, Waltham, KS, USA). The Raman spectra of the C-dots were tested by a micro confocal Raman spectrometer (Raman, inVia, Renishawin, Gloucestershine, UK).

### 2.4. Detection of Pyridine

First, 99% pure pyridine was diluted to prepare a 0.01 mol/L pyridine solution. To prepare 1 mg/mL C-dots stock solution, we weighed 10 mg of C-dots powders and put them into a 25 mL glass bottle, following which 10 mL of methanol was added. After that, 0.5 mL of the C-dots stock solution was diluted with methanol (49.5 mL) to generate the 10 μg/mL C-dots solution. Then 3 mL of the C-dots solution (10 μg/mL) was put into a 1 cm × 1 cm × 3 cm quartz cuvette, and the UV–vis absorption and PL emission spectrum of the C-dots without any analyte were tested. To detect pyridine, 10 μL of pyridine solution (0.01 mol/L) was added to the C-dots cuvette and mixed well to observe the color change; following that, the UV–vis absorption and PL emission spectrum of the mixed solution were also measured.

### 2.5. Solution Assays

3 mL of pyridine solutions of different concentrations were transferred to 1 cm × 1 cm × 3 cm quartz cuvettes, and then 15 μL of C-dots (1 mg/mL) solutions were added to these cuvettes. After fully mixed, the colors of the mixed solutions were observed and recorded. The sensing of other NCOBs followed the same procedures.

### 2.6. Test Paper Assays

1 mL of the C-dots stock solution (1 mg/mL) was diluted with 9 mL of methanol to make the 0.1 mg/mL C-dots solution. Then cut test papers (1.5 cm × 4 cm) were soaked in the C-dots solution for 20 mins to make the C-dots test papers. After that, NCOBs of different concentrations were dropped onto the test papers to observe the color change.

### 2.7. Spectral Sensing Assays Based on UV–vis Absorption

0.5 mL of the C-dots stock solution (1 mg/mL) was diluted with 49.5 mL of methanol to prepare the 10 μg/mL C-dots solution. Then the C-dots solutions (10 μg/mL, 1.5 mL) were transferred to quartz cuvettes (1 cm × 1 cm × 3 cm) and mixed with 1.5 mL of NCOBs of different concentrations. After shaking well, the UV-vis absorptions of the mixed solutions were recorded, and calibration curves relating the intensities of absorbance at 626 nm and the concentrations of the NCOBs were established.

### 2.8. Spectral Sensing Assays Based on Fluorescence Spectroscopy

0.5 mL of the C-dots stock solution (1 mg/mL) was diluted with 49.5 mL of methanol to prepare the 10 μg/mL C-dots solution. Then the C-dots solutions (10 μg/mL, 1.5 mL) were transferred to quartz cuvettes (1 cm × 1 cm × 3 cm) and mixed with 1.5 mL of NCOBs of different concentrations. After shaking well, the fluorescence emissions of the mixed solutions excited at 540 nm were recorded, and then calibration curves relating the intensities of fluorescence emissions at 602 nm and the concentrations of the NCOBs were established.

### 2.9. Difference in Percentage (Diff. %) between Calculated Values and Actual Values

The 3.0 nM pyridine solution was prepared by diluting the pyridine solution of 6.0 nM. Specifically, 1.5 mL of the pyridine solution (6.0 nM) and 1.5 mL of C-dots (10 μg/mL) solution were transferred to a 1 cm × 1 cm × 3 cm quartz cuvette, mixed well and the absorption spectrum was tested with a UV-vis spectrometer (or emission spectrum with fluorescence spectrophotometer). The absorption intensity at 626 nm of the absorption spectrum (or emission intensity at 602 nm in the PL spectrum) was taken into the respective calibration curves of pyridine obtained above to calculate the concentration of pyridine (c). The Diff. % of the respective assay for sensing pyridine was then calculated following the formula

(1) $$\mathrm{Diff}.\;\%=\frac{\left|c-3\right|}{3}\text{×}100\%$$

The Diff. % calculations for pyridine in different concentrations (9.0 and 17.0 nM) as well as other NCOBs followed the same procedure for 3.0 nM pyridine solution described here.

## 3. Experimental Results and Discussion

### 3.1. Synthesis and Characterization of C-Dots

**Figure 2:**
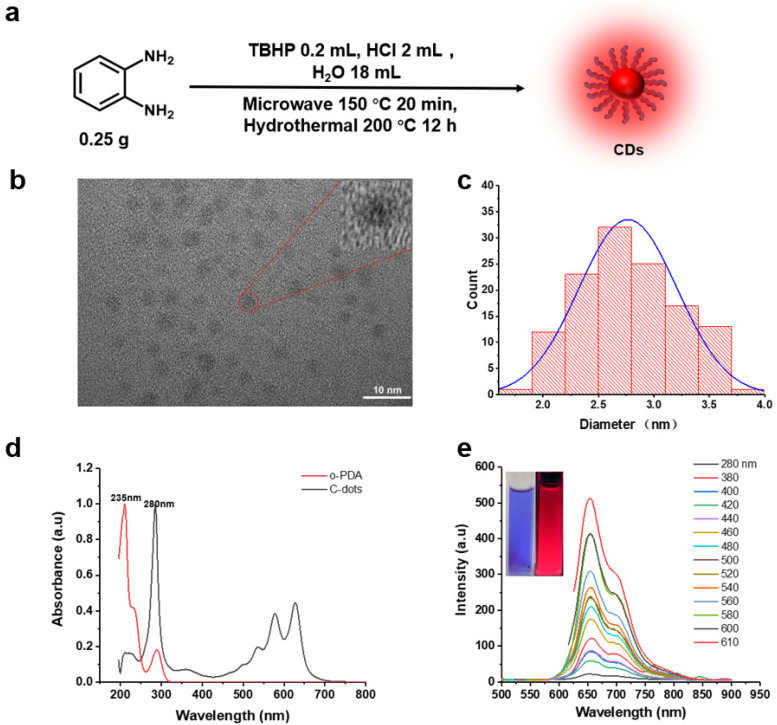
(**a**) Synthesis route of C-dots. (**b**) TEM image (the inset is an enlarged view of a particle) of the C-dots particles. (**c**) Size distribution histogram of C-dots. (**d**) UV–vis spectral of C-dots vs. the carbon precursor o-PDA. (**e**) PL spectral of C-dots excited at various wavelengths. The inset: Photographs of C-dots under ambient light (left) and 365 nm handheld UV light (right).

The C-dots used in this study were synthesized from o-PDA in an acidic (HCl) and oxidizing (TBHP) environment (Figure 2a), the obtained sample was blackish powder. We carried out various microscopic and spectroscopic analytical methods to study the morphology and chemical compositions of the C-dots. First, the morphological and structural characteristics of C-dots were explored using TEM microscopy. TEM shows that these C-dots are uniformly dispersed spherical particles without obvious aggregation (Figure 2b), which could be attributed to the strong mutual repulsions among these particles due to their high surface charges as indicated by their zeta potential (+30.1 mV). A close look at these particles reveals that they have no obvious lattice structures (Figure 2b, inset), indicating that they are amorphous rather than graphite structures. Overall, these particles range from 1.81–3.68 nm in diameters, with an average of 2.77 nm (Figure 2c).

Next, we studied the spectroscopic behaviors of the obtained C-dots. The absorption peaks at 235 and 280 nm in the UV–vis spectral are attributed to the n-π* transition of C-NH_2_, and the π-π* transition of C=C/C=N, respectively; while the peaks above 300 nm are attributed to core absorptions of C-dots [14,15] (Figure 2d). Compared to the carbon precursor (o-PDA), it is obvious that the UV–vis absorption peak of C-dots at 235 nm significantly decreased, while that at 280 nm greatly increased. This could be attributed to the consumption of C-NH_2_, and formation of C=C/C=N functionalities in the polymerization and carbonization processes during the formation of C-dots. We then studied the PL behavior of the C-dots. It is interesting to see, unlike most of C-dots reported so far, our sample demonstrated fluorescence emissions that are independent of their excitation wavelengths. Despite the alteration of the excitation wavelengths, our C-dots always demonstrate an emission spectrum with a main peak at 655 nm, and a shoulder at 700 nm that further extends to as far as 850 nm (Figure 2e). As a result, the blue transparent solution of C-dots under ambient light (left, Figure 2e inset) readily turns into a deep red solution when excited by a 365 nm handheld UV lamp (right, Figure 2e inset).

**Figure 3:**
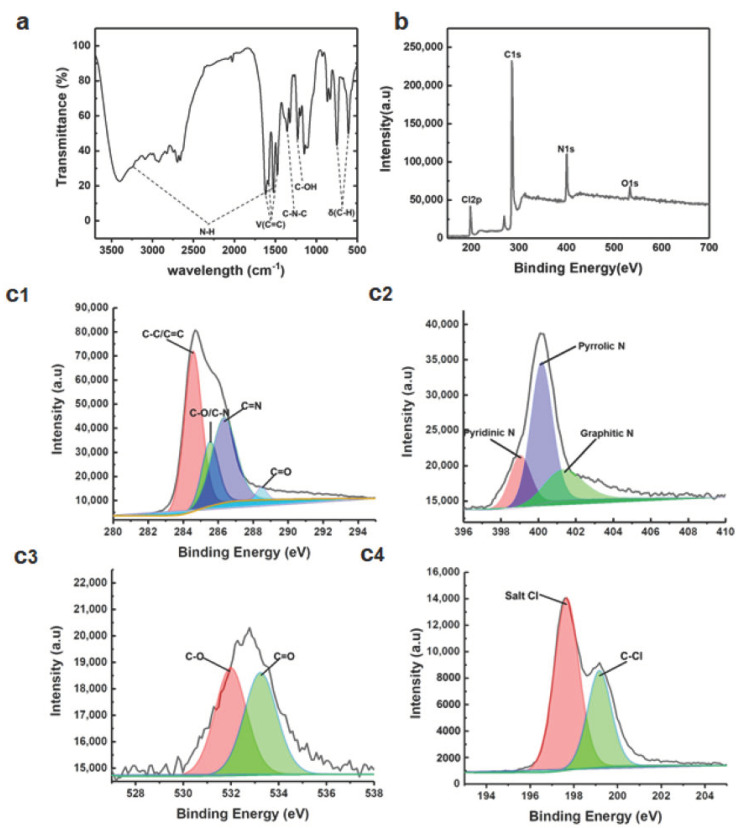
(**a**) FTIR spectrum of C-dots. (**b**) XPS survey spectrum of C-dots. (**c1**) High-resolution C1s XPS. (**c2**) High-resolution N1s XPS. (**c3**) High-resolution O1s XPS. (**c4**) High-resolution Cl XPS.

In order to further study the chemical compositions and surface functionalities of the C-dots, FTIR, and XPS spectroscopy were performed (Figure 3). In the FTIR spectrum, the absorption peaks at 3242 and 1520 cm^−1^ are characteristic peaks of amino functional groups. 1621, 1586, and 1468 cm^−1^ are the characteristic absorption peaks of C=C form benzene ring structure. 1364 cm^−1^ is attributed to the C-N-C characteristic absorption peak of the phenazine structure [16] while 1236 cm^−1^ is the absorption peak caused by the vibration of the C-OH structure. 605 and 745 cm^−1^ are the absorption peaks caused by the C-H vibration of the phenazine structure (Figure 3a). These functionalities are in accordance with studies in which o-PDA was used as starting materials for the construction of C-dots [16]. FTIR spectroscopy shows that C-dots have abundant amine, hydroxyl, and phenazine moieties. XPS spectroscopy further confirms these functionalities. The measured spectrum (Figure 3b) shows that C-dots are mainly composed of C (78.25%), N (14.38%), O (3.19%), and Cl (4.19%). The high-resolution C spectrum (Figure 3c1) shows four different types of C: C-C/C=C (284.56 eV), C-O/C-N (285.55 eV), C=N (286.37 eV), and C=O (288.5 eV) [17]. This further supports that C-dots contain phenazine and amino moieties. The high-resolution N spectrum (Figure 3c2) shows three peaks at 399.21 eV, 400.32 eV, and 401.36 eV corresponding to pyridine, pyrrole and graphitic nitrogen, respectively [15]. The high-resolution O spectrum (Figure 3c3) shows two different types of O: C-O (531.62 eV), C=O (533.48 eV), which indicates that the surfaces of C-dots contain some amount of hydroxyl and carboxyl groups. Two distinct peaks, C-Cl (199.22 eV) and salt Cl (197.64 eV) were observed (Figure 3c4), indicating that C-dots formed a salt, which might be attributed to that the amino and pyridine nitrogens on the surface of C-dots were protonated during the reaction by HCl.

### 3.2. Colorimetric Sensing Assay Development

**Figure 4:**
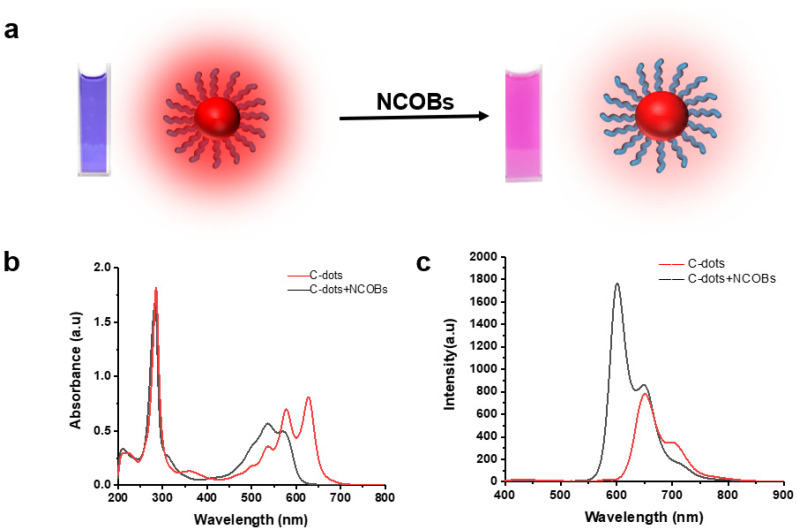
Alternations of C-dots in the presence of NCOBs (pyridine): (**a**) Color change of C-dots solution; (**b**) UV–vis absorption change of C-dots; and (**c**) PL emission change of C-dots.

Optical sensing refers to the sensing of analytes based on optical principles and signals. It has many advantages such as non-contact and non-destructive measurement, high-speed transmission, streamlined testing, etc. Depending on the nature of the optical signals, common optical sensors include colorimetric sensors and spectral sensors. To our delight, we found that the C-dots are very sensitive to NCOBs: in the presence of NCOBs (i.e., pyridine), the solution of C-dots has a dramatic visual change, turning from bright blue into deep red (Figure 4a), which is very useful for the development of colorimetric sensors. Furthermore, our study found that both the UV–vis absorption and PL of C-dots had characteristic alternations in the presence of NCOBs. For instance, there is an overall blue shift of the UV–vis absorption, and the absorption at 626 nm of the C-dots could eventually disappear in the presence of pyridine (Figure 4b). A blue shift of the best emission peak in the PL spectrum of C-dots from 655 nm to 606 nm was also observed in the presence of pyridine; and the emissions were greatly enhanced (Figure 4c).

**Figure 5:**
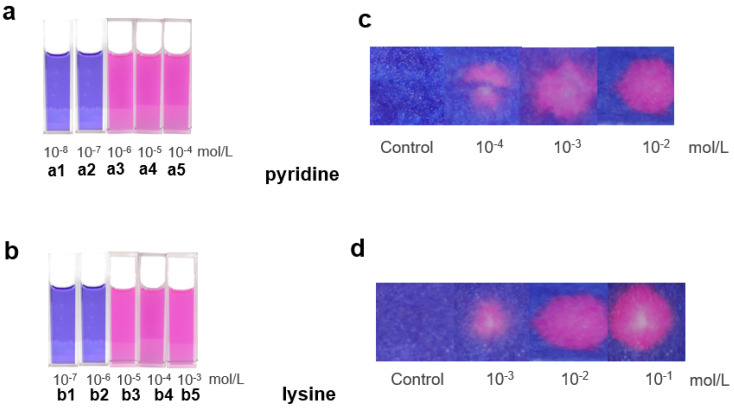
Figures showing the color alternations of C-dots solutions in presence of (**a**) pyridine and (**b**) lysine of different concentrations. Figures showing the color alternations of test papers when dropped with (**c**) pyridine and (**d**) lysine of different concentrations.

Based on the above-mentioned findings, we began to test the possibility to develop assays for the sensing of NCOBs both colorimetrically and optically. Taking pyridine as an example, we first aimed to develop a simple and straightforward sensing assay for the detection of NCOBs based on visual changes. Interestingly, the color change of C-dots actually depends on the concentrations of NCOBs. Our study shown that there was no observable color change of the C-dots solution when the concentration of pyridine is too low (less than 10^−7^ M, Figure 5(a1,a2)); however, when the concentration of pyridine reaches 10^−6^ M, it could turn the C-dots solution from bright blue to deep red. In fact, any pyridine solution higher than 10^−6^ M could change the color of the C-dots, and the higher the concentration, the more obvious the color changes (Figure 5(a3–a5)). We then tested lysine, a commonly seen amino acid that plays important roles in protein synthesis and regulation of human growth factors, and observed similar trend: the color of C-dots solution would not change when concentrations of lysine were low (less than 10^−6^ M, Figure 5(b1,b2)), however, as long as the concentrations of lysine were higher than 10^−5^ M, it could readily turn the C-dots into deep red. The color of C-dots solutions became much deeper as the concentrations increased (Figure 5(b3–b5)). To our delight, we found that all the NCOBs mentioned above (Figure 1) could be detected using this C-dots solution-based assay (Figure S1). It is worth to mentioning, however—depending on the basicity of the NCOBs—that the minimum concentrations required for a successful visual detection are different, which have been summarized and presented in Table 1. As can be seen, the highest minimum concentration required for a successful visual detection is pyrrole, which could be attributed to its weak basicity (pKa of the conjugated acid of pyrrole = 0.41); on the other hand, for spermine, thanks to its strong basicity (pKa of the conjugated acid of spermine = 10.88), the minimum concentration required for a successful visual detection is as low as 10^−6^ M.

Although the solution assay demonstrated high sensitivity and convenience for the detection of NCOBs; however, solutions are not convenient to transport, store, and use in practical applications. Inspired by traditional pH test papers, we moved ahead to test the possibility of making a test paper version of this solution assay. Excitingly, we were able to fulfill this aim, and the test paper version of this assay is fully functional. Simply dropping sample solutions onto the test paper and a color change would indicate the presence of NCOBs. We found that the test paper assay behaved very similar to the solution assay. For instance, only pyridine solutions with concentrations higher than 10^−4^ M could turn the test paper into red; and the higher the concentration of pyridine, the deeper the color of test paper become (Figure 5c). Again, we tested lysine and obtained similar results; the only difference is that the minimum concentration required for a successful detection is slightly higher, reaching 10^−3^ M (Figure 5d). To our delight, the test paper assay was able to detect all the NCOBs discussed in the solution assay (Figure S2). Depending on the basicity of the NCOBs, the minimum concentrations required for a successful visual detection using the test paper are different, which have also been summarized and presented in Table 1. From Table 1 we could see that the minimum concentrations required for test paper detection are higher than those required in solution assays. However, the test paper assay has its advantages, such as ease in storage, transportation, and usage.

### 3.3. Spectral Sensing Assay (UV-vis and PL) Development

**Figure 6:**
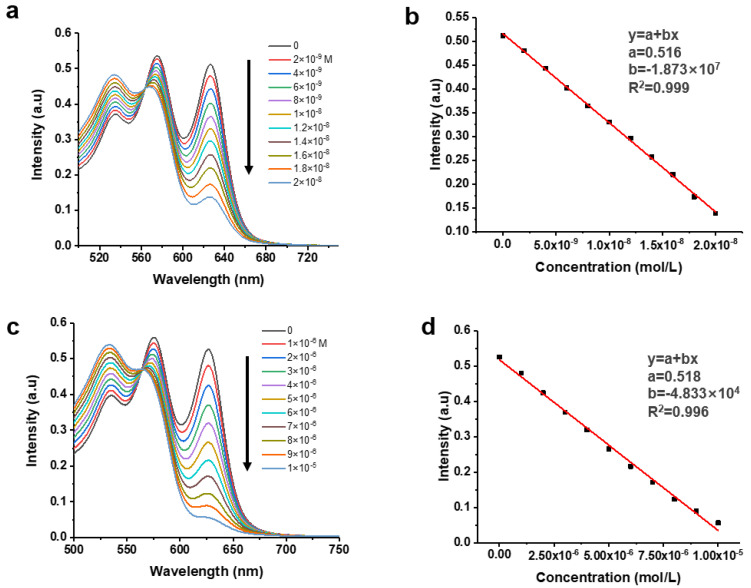
(**a**) Alternations of the UV–vis spectral of C-dots (10 μg/mL) in presence of pyridine solutions of different concentrations. (**b**) Calibration curve showing the linear relationship between the absorption intensities of C-dots at 626 nm and the concentrations of pyridine. (**c**) Alternations of the UV–vis spectral of C-dots (10 μg/mL) in presence of lysine solutions of different concentrations. (**d**) Calibration curve showing the linear relationship between the absorption intensities of C-dots at 626 nm and the concentrations of lysine.

The solution and paper assays are good for the quick screening of solutions to qualitatively confirm the presence of NCOBs; however, their sensitivities are relatively low and in some occasions quantitative detections are required. In this context, we began to seek the possibility to develop optical assays that are much more sensitive and able to detect NCOBs quantitatively. As discussed above, the absorption at 626 nm of the C-dots would decrease and eventually disappear in the presence of NCOBs (Figure 4b). Thus, we studied the alternations of this absorption in the presence of various concentrations of pyridine. As shown in Figure 6a, the absorption of C-dots at 626 nm gradually decreased with increased concentration of pyridine. Clearly, the spectral establish a negative correlation between the concentrations of NCOBs (pyridine) and the UV-vis absorption signal intensities of C-dots, indicating that the determination of pyridine concentration using this C-dots assay is feasible.

On the basis of the analysis, a calibration curve correlating concentrations of pyridine with the UV-vis absorption intensities at 626 nm of C-dots was successfully established (Figure 6b). As shown in the graph, the concentrations of pyridine have an excellent linear relationship (the correlation coefficient R^2^ is 0.999) with the absorption intensities of C-dots at 626 nm. According to well-accepted 3σ method [18,19], the LOD of this assay was determined to be 0.75 nM. When we further increased the concentration of pyridine (from 0 nM to 20 nM), we observed the saturation of signals when pyridine concentration is larger than 20 nM; however, the assay has very good linearity when pyridine concentration is less than 20 nM. The limit of quantitation (LOQ) of an assay is defined as the threshold at which the calibration curve could be confidently used to determine the concentration of an analyte. According to well-accepted 10σ method [18,19], the LOQ of this assay was determined to be 2.27 nM, thus the linear range of this assay is from 2.27 to 20 nM. We also tested lysine and found that concentrations of lysine and intensities of absorptions of C-dots at 626 nm also had a good correlation relationship (Figure 6c). Based on the calibration curve (Figure 6d), the LOD and linear range of this assay for the sensing of lysine were determined to be 0.29 μM and 0.88 to 10 μM, respectively.

We were glad to find that similar results were observed when other NCOBs presented in Figure 1 were studied: good correlations between the concentrations of the NCOBs and the absorption intensities of C-dots at 626 nm were established and calibration curves were constructed (Figures S3–S11). Key parameters for the detection of NCOBs using this UV-vis absorption-based optical assay are summarized in Table 2. From the table, we could see that the basicity of the NCOBs has significant influence on the sensitivity of this assay: NCOBs with high basicity (i.e., spermine, aniline, as well as pyridine) generally have the lowest LODs that are less than 1.0 nM; while for pyrrole, which has the weakest basicity, its LOD is much higher (0.12 mM).

**Figure 7:**
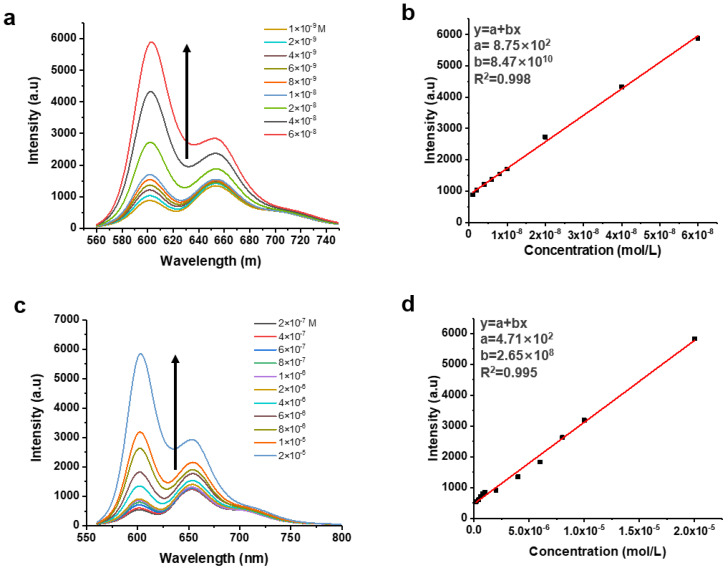
(**a**) Alternations of the PL spectral of C-dots (10 μg/mL) in presence of pyridine solutions of different concentrations. (**b**) Calibration curve showing the linear relationship between the PL intensities of C-dots at 606 nm and the concentrations of pyridine. (**c**) Alternations of the PL spectral of C-dots (10 μg/mL) in presence of lysine solutions of different concentrations. (**d**) Calibration curve showing the linear relationship between the PL intensities of C-dots at 606 nm and the concentrations of lysine.

With the successful establishment of UV-vis absorption-based sensing assay, we moved ahead to the development of sensing assay based on the PL alteration of C-dots as demonstrated in Figure 4c. As expected, C-dots demonstrated an obvious blue shift in PL from 655 to 606 nm in presence of pyridine; and the PL intensities at 606 nm gradually increased as the concentrations of pyridine became higher (Figure 7a). Based on this observation, a calibration curve correlating concentrations of pyridine with the PL intensities at 606 nm of C-dots was successfully established (Figure 7b). As demonstrated, concentrations of pyridine have an excellent linear relationship (correlation coefficient R^2^ is 0.998) with the PL intensities of C-dots at 606 nm. Furthermore, the LOD, LOQ, and linear range of this assay were determined to be 0.68 nM, 2.06 nM, and 2.06 to 60.00 nM, respectively. We tested lysine and similar results were observed (Figure 7c), using the calibration curve established (Figure 7d), LOD, LOQ, and linear range of this assay for the sensing of lysine were determined to be 0.20 μM, 0.61 μM, and 0.61 to 20 μM, respectively. To our delight, good correlations between the concentrations of all the NCOBs previously discussed and the PL intensities of C-dots (at 606 nm) were established and calibration curves were constructed (Figures S12–S20). Key parameters for the detection of NCOBs using this PL-based optical assay are summarized in Table 3. Similarly, the sensitivity of this PL-based assay also largely depends on the basicity of the NCOBs tested. NCOBs with stronger basicity generally resulted in high sensitivity.

### 3.4. Reliability and Selectivity of the Assay

To probe the reliability of the assays developed, we made spike solutions of the eleven NCOBs we studied. Then both the UV-vis absorption- and PL-derived calibration curves were used to quantify the spike solutions and compared to the actual concentrations. To our delight, the UV-vis absorption-based assay seems very reliable (column 4 in Table 2): for all NCOBs tested, the differences between the calculated values and the actual values are all within 8%, and the lowest (for spermine) is less than 0.9%. As expected, the PL-based assay is also very reliable (column 4 in Table 3): for all NCOBs detected, the differences between the calculated values and the actual values are all within 7%.

**Figure 8:**
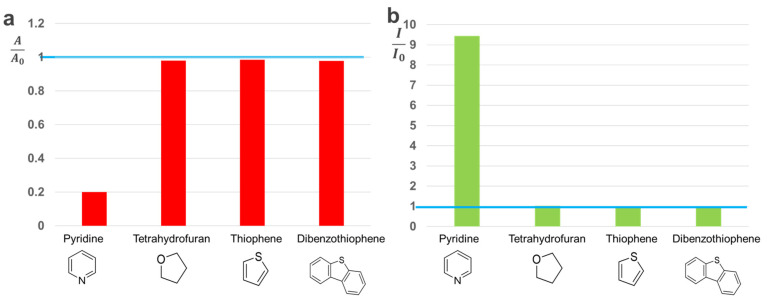
Selectivity of the NCOBs sensing assay towards oxygen and sulfur containing compounds: (**a**) UV-vis absorption-based assay, where A_0_ is the UV-vis absorption intensity of C-dots at 626 nm without any analytes, and A is the UV-vis absorption intensity of C-dots at 626 nm in the presence of 10^−3^ M analytes. (**b**) PL-based assay, where I_0_ is the PL intensity of C-dots at 606 nm without any analytes, and I is the PL intensity of C-dots at 606 nm in the presence of 10^−3^ M analytes.

In addition to the high sensitivity and reliability, to our delight, the assay developed above also demonstrated excellent selectivity towards different types of similar organics. Specifically, the assay has an excellent ability to distinguish NCOBs from oxygen and sulfur containing organics. In most application scenarios that need to detect NCOBs, oxygen- and sulfur-containing organics are the main interfering factors for detection. For example, to determine whether wastewater meets the discharge requirements, one of the main criteria is whether NCOBs meet the standards. In agricultural fertilizers, NCOBs are the main criteria for judging their quality. However, oxygen- and sulfur-containing organics are generally co-presented in these scenarios, thus the exclusion of their interference would be key points to consider when designing such sensing assays. To our delight, oxygen and sulfur-containing matters (i.e., tetrahydrofuran, thiophene, and dibenzothiophene) hardly caused any changes to the color of the C-dots solution and test paper (data not shown) in our study; furthermore, these organics were also not able to cause any significant changes to the UV-vis and PL of C-dots that might interfere with the sensing of NCOBs (Figure 8). As can be seen, in the presence of NCOBs (i.e., pyridine), both the UV-vis absorption (decreased, Figure 8a) and the PL (enhanced, Figure 8b) of C-dots were significantly altered as described above. However, neither UV-vis absorption nor PL of C-dots had observable changes in the presence of oxygen or sulfur containing organics. Therefore, the assay developed here has potential applications such as determination of nitrogen contents in nitrogen fertilizers in agricultural production, as well as the detection of nitrogen-containing organic pollutants in wastewater where oxygen and sulfur containing molecules are generally co-presented.

### 3.5. Advantages of the Assay

It is worth mentioning that, compared to the previous methods, the assay developed in this work has obvious advantages: (1) due to the complicated design strategies, the sporadic assays reported could only cover one specific NCOBs [20,21]. Thus, one has to switch among several completely different assays to detect different NCOBs targets, which is not only troublesome but also raises the costs significantly. However, this is not an issue for our assay as it responds well to most NCOBs (and not limited to examples we tested). (2) In addition to its broad scope, our assay is also very sensitive: out of the 11 example NCOBs we tested, 2 of them have never been achieved in literature before; and 5 of them are much better in terms of their sensitivity (or LOD) than the best results reported in literature (Table S2) [22,23,24,25,26]. (3) The assay could distinguish NCOBs from oxygen and sulfur containing organics very well. (4) The design and construction of our assay is straightforward, which does not require any modifications or composition to the sensing platform (C-dots).

## 4. Conclusions

In this study, we have developed both colorimetric (including solution and test paper type) and spectral sensors (including UV–vis and PL type) for the quick and sensitive detection and determination of general NCOBs, which include aliphatic amines (e.g., butylamine, spermine), aromatic amines (e.g., aniline), five-membered heterocyclics (e.g., pyrrole, imidazole), six-membered heterocyclics (e.g., pyridine), fused-ring heterocyclics (e.g., indole, quinolone, purine), amino acids (e.g., lysine), and antibiotics (e.g., tetracycline). The colorimetric sensing assays (solution and test paper assays) respond quickly and are easy to use; while for spectral (UV–vis and PL) assays, they provided quick accesses for the sensitive detection of NCOBs, LODs of which are as low as 0.5 nM. Most importantly, these assays demonstrated high reliability, for all NCOBs detected, the differences between the calculated values and the actual values are all within 8%. The sensor also demonstrated excellent ability to distinguish NCOBs from oxygen and sulfur containing organics. In summary, our assay in this work for the sensing of NCOBs has the advantages of broad sensing scope, high sensitivity and selectivity, easy operation and fast sensing speed. Thus, the assay developed here should find potential applications in monitoring the nitrogen contents of nitrogen fertilizers in agricultural production and estimating nitrogen-containing organic matters in wastewater.

## Figures and Tables

**Table 1:** Sensitivities of solution and test paper assays developed from C-dots for the detection of various NCOBs.

	NCOBs	Aliphatic Amines		Aromatic	Five-H ^a^		Six-H ^b^	Fused Ring			Amino Acid	Antibiotic
Assays		Butylamine	Spermine	Aniline	Pyrrole	Imidazole	Pyridine	Purine	Quinoline	Indole	Lysine	Tetracycline
Solution (M)		10^−5^	10^−6^	10^−6^	10^−2^	10^−5^	10^−6^	10^−3^	10^−3^	10^−2^	10^−5^	10^−5^
Paper (M)		10^−4^	10^−6^	10^−5^	10^−1^	10^−4^	10^−4^	10^−1^	10^−2^	10^−1^	10^−3^	10^−4^

^a^ Five-membered heterocyclic compound. ^b^ Six-membered heterocyclic compound.

**Table 2:** Key parameters for the detection of NCOBs using UV-vis based, C-dots derived sensing assays.

NCOBs	LOD ^a^	Linear Range	Diff. % ^b^	Figure ^c^
butylamine	5.50 × 10^−2^ µM	0.17–3 µM	1.00	S3
spermine	0.70 nM	2.12–20 nM	0.90	S4
aniline	0.69 nM	2.06–20 nM	2.77	S5
pyrrole	0.12 mM	0.36–2 mM	1.87	S6
imidazole	7.40 × 10^−2^ µM	0.22–1.60 µM	1.73	S7
pyridine	0.75 nM	2.27–20 nM	2.15	6a,b
purine	0.042 mM	0.13–0.30 mM	7.67	S8
quinoline	0.015 mM	0.045–0.20 mM	5.43	S9
indole	0.025 mM	0.076–0.20 mM	5.07	S10
lysine	0.29 µM	0.88–10 µM	1.41	6c,d
tetracycline	0.85 µM	2.58–20 µM	3.90	S11

^a^ LOD, limit of detections (3σ). ^b^ Diff. %: Differences between calculated values and actual values in percentage. The data presented is average of three testings at different concentrations; for details, please see Table S1 in the supporting information. ^c^ Respective spectral and calibration curves from which the data presented were calculated.

**Table 3:** Key parameters for the detection of NCOBs using PL-based, C-dots derived sensing assays.

NCOBs	LOD ^a^	Linear Range	Diff. % ^b^	Figure ^c^
butylamine	0.17 µM	0.50–20 µM	5.03	S12
spermine	0.50 nM	1.52–100 nM	3.47	S13
aniline	0.53 nM	1.61–10 nM	6.20	S14
pyrrole	1.55 mM	4.70–100 mM	6.10	S15
imidazole	0.17 µM	0.52–100 µM	5.10	S16
pyridine	0.68 nM	2.06–60 nM	6.77	7a,b
purine	12.40 µM	37.60–100 µM	6.00	S17
quinoline	17.40 µM	52.70–1000 µM	5.13	S18
indole	87.10 uM	0.26–1 mM	4.80	S19
lysine	0.20 µM	0.61–20 µM	6.00	7c,d
tetracycline	0.20 µM	0.60–10 µM	4.17	S20

^a^ LOD, limit of detections (3σ). ^b^ Diff. %: Differences between calculated values and actual values in percentage. The data presented is are averages of three testings at different concentrations; details please see Table S1 in the supporting information. ^c^ Respective spectral and calibration curves from which the data presented were calculated.

## Data Availability

The data presented in this study are available within the article and supplementary material.

## Supplementary Materials

The following are available online at https://www.mdpi.com/article/10.3390/nano11102607/s1, Figure S1: Solution assays for the detection of nitrogen-containing organic bases (NCOBs); Figure S2: Test paper assays for the detection of NCOBs; Figure S3: Detection of butylamine using UV-vis absorption-based sensing assay; Figure S4: Detection of spermine using UV-vis absorption-based sensing assay; Figure S5: Detection of aniline using UV-vis absorption-based sensing assay; Figure S6: Detection of pyrrole using UV-vis absorption-based sensing assay; Figure S7: Detection of imidazole using UV-vis absorption-based sensing assay; Figure S8: Detection of purine using UV-vis absorption-based sensing assay; Figure S9: Detection of quinoline using UV-vis absorption-based sensing assay; Figure S10: Detection of indole using UV-vis absorption-based sensing assay; Figure S11. Detection of tetracycline using UV-vis absorption-based sensing assay; Figure S12: Detection of butylamine using PL-based sensing assay; Figure S13: Detection of spermine using PL-based sensing assay; Figure S14: Detection of aniline using PL-based sensing assay; Figure S15: Detection of pyrrole using PL-based sensing assay; Figure S16: Detection of imidazole using PL-based sensing assay; Figure S17: Detection of purine using PL-based sensing assay; Figure S18: Detection of quinoline using PL-based sensing assay; Figure S19: Detection of indole using PL-based sensing assay; Figure S20: Detection of tetracycline using PL-based sensing assay; Table S1: Summary of differences between calculated values and the actual values in percentage (Diff. %) of UV-vis absorption- and PL-based assays for the detection of various NCOBs; Table S2: Comparison of the performance of NCOBs sensing reported in this work to that of prior art [22,23,24,25,26,27,28,29,30].
