# Supplementary material for: Facile and Sensitive Detection of Nitrogen-Containing Organic Bases with Near Infrared C-Dots Derived Assays
Source: Nanomaterials (Basel). 2021 Oct 3;11(10):2607. doi: 10.3390/nano11102607 (PMC8537226; doi:10.3390/nano11102607)
Supplement: Supplementary file 1 [file nanomaterials-11-02607-s001.zip › nanomaterials-1394293-supplementary.pdf]

Supplementary Materials

Facile and Sensitive Detection of Nitrogen-Containing Organic Bases with Near Infrared C-Dots Derived Assays

Chunyu Ji [1], Yiqun Zhou [2], Wenquan Shi [1], Jiajia Wu [1], Qiurui Han [1], Tianshu Zhao [1], Roger M. Leblanc [2] and Zhili Peng [1,*]

1. National Center for International Research on Photoelectric and Energy Materials, School of Materials and Energy, Yunnan University, Kunming 650091, China; jichunyu@mail.ynu.edu.cn (C.J.); shiwenquan1010@163.com (W.S.); 12019101477@mail.ynu.edu.cn (J.W.); hqr3155064793@163.com (Q.H.); zhaotianshu@mail.ynu.edu.cn (T.Z.)
2. Department of Chemistry, University of Miami, 1301 Memorial Drive, Coral Gables, FL 33146, USA; yxz431@miami.edu (Y.Z.); rml@miami.edu (R.M.L.)
3. Correspondence: zhilip@ynu.edu.cn; Tel.: +86–871–65037399

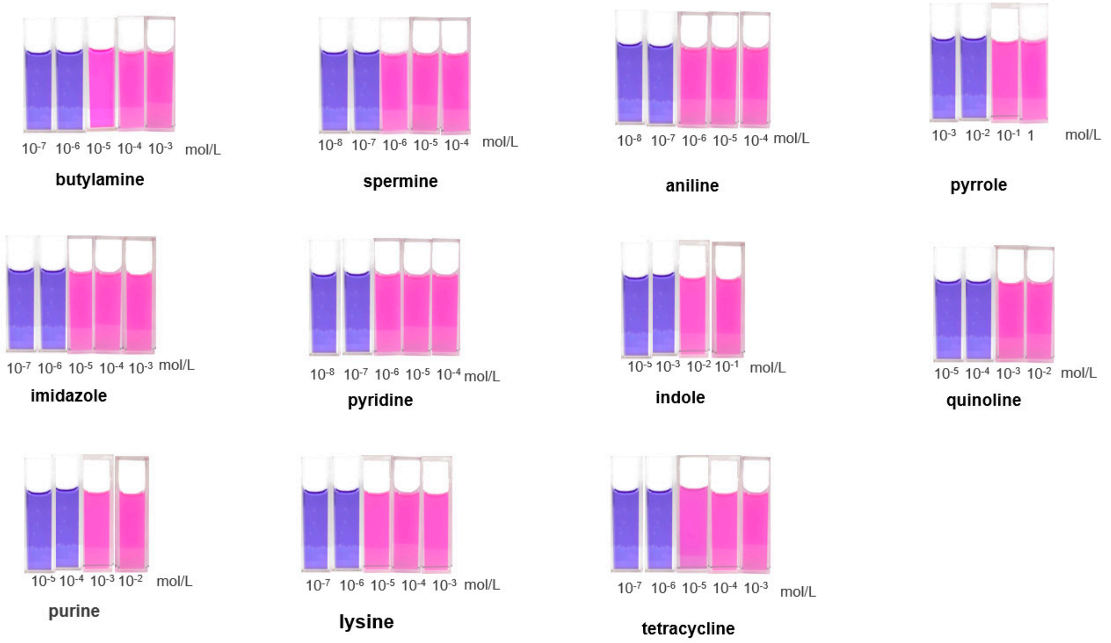

Figure S1. Solution assays for the detection of nitrogen-containing organic bases (NCOBs)

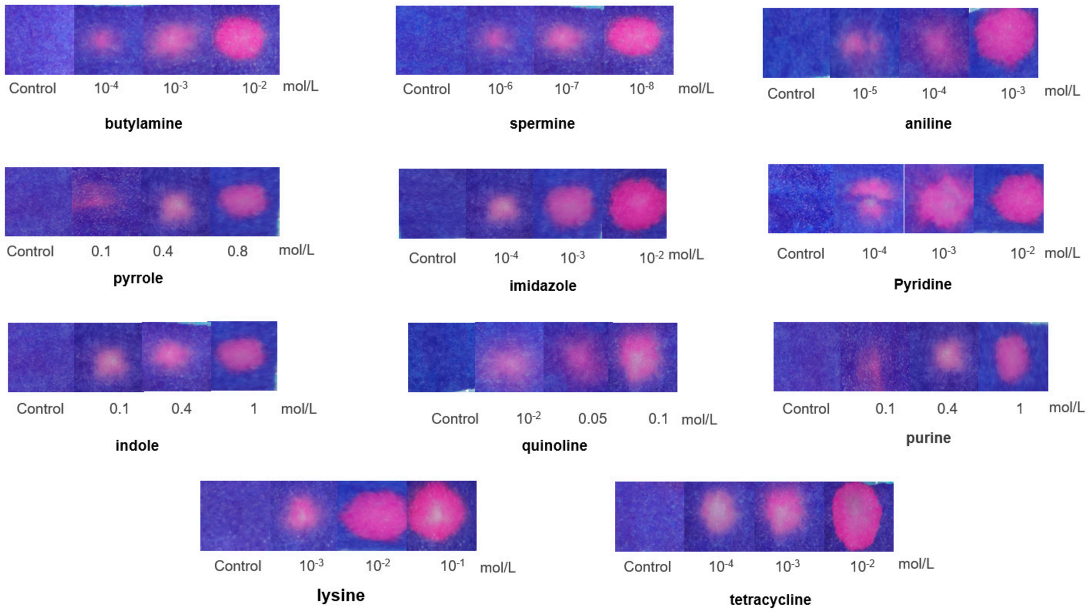

Figure S2. Test paper assays for the detection of NCOBs

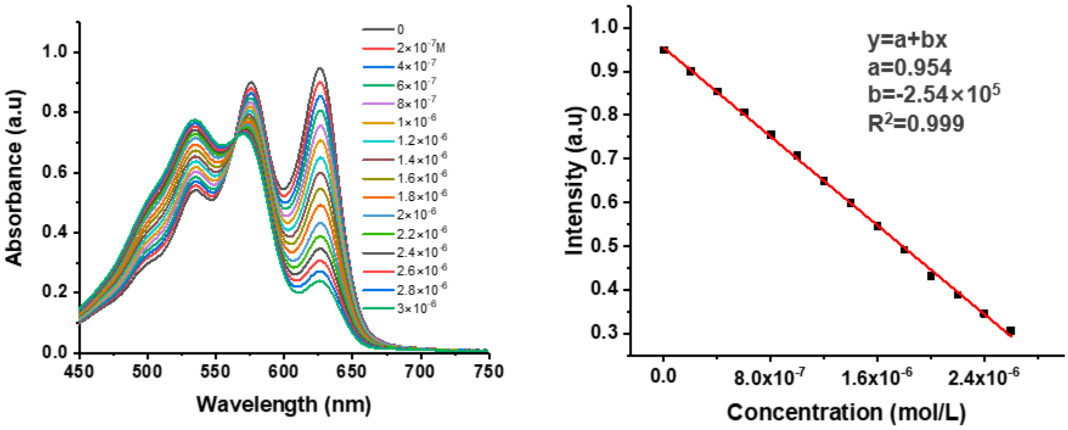

Figure S3. Detection of butylamine using UV-vis absorption-based sensing assay

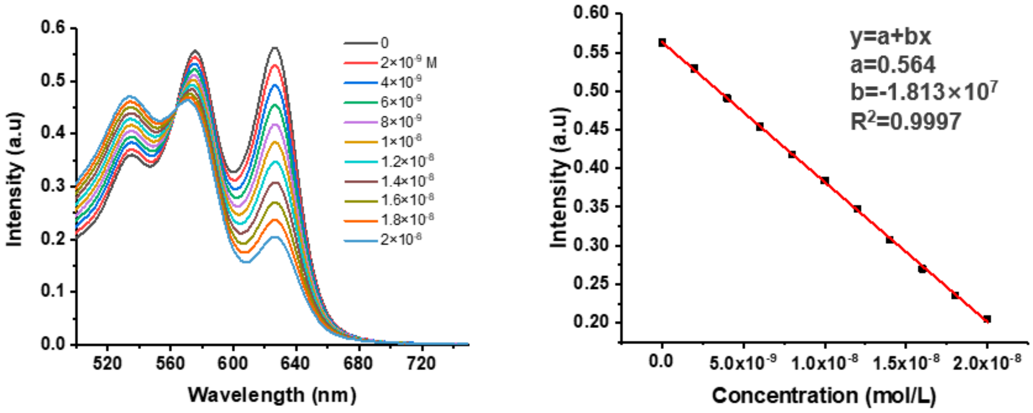

Figure S4. Detection of spermine using UV-vis absorption-based sensing assay

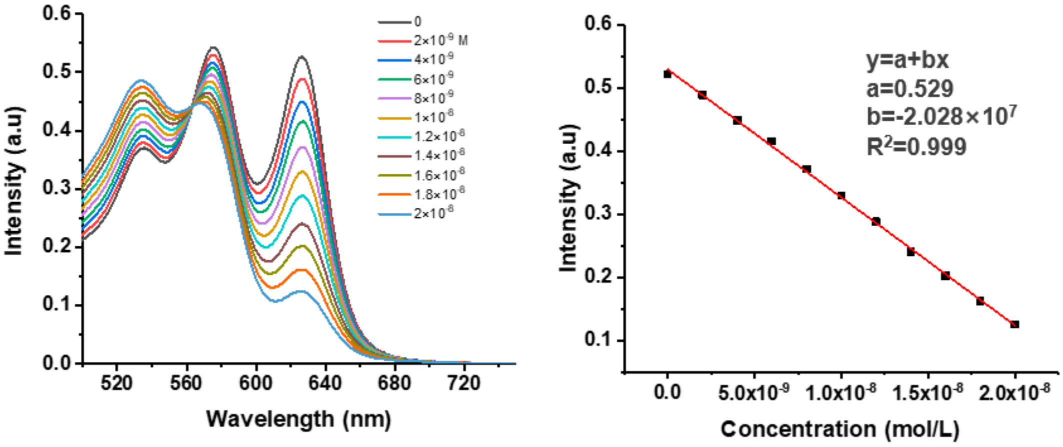

Figure S5. Detection of aniline using UV-vis absorption-based sensing assay

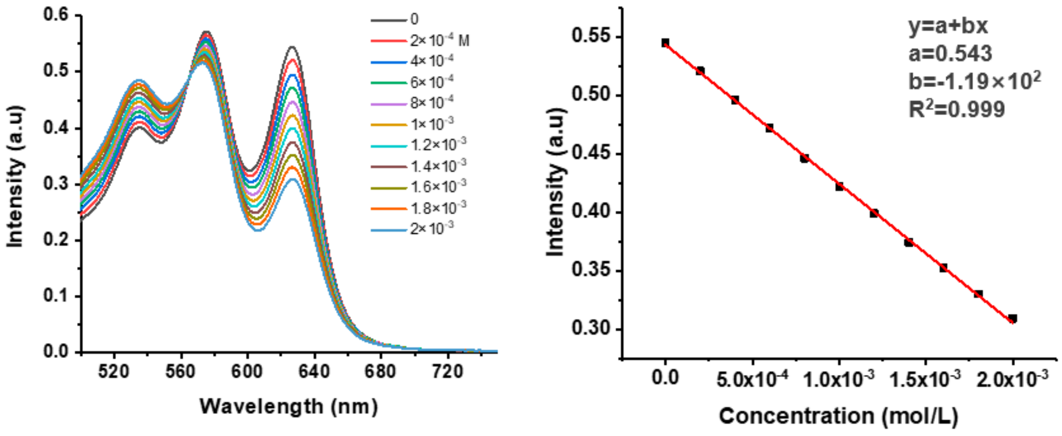

Figure S6. Detection of pyrrole using UV-vis absorption-based sensing assay

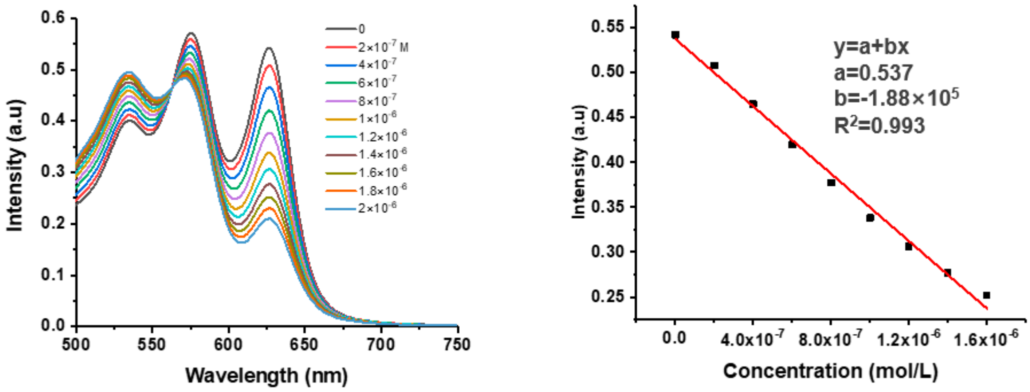

Figure S7. Detection of imidazole using UV-vis absorption-based sensing assay

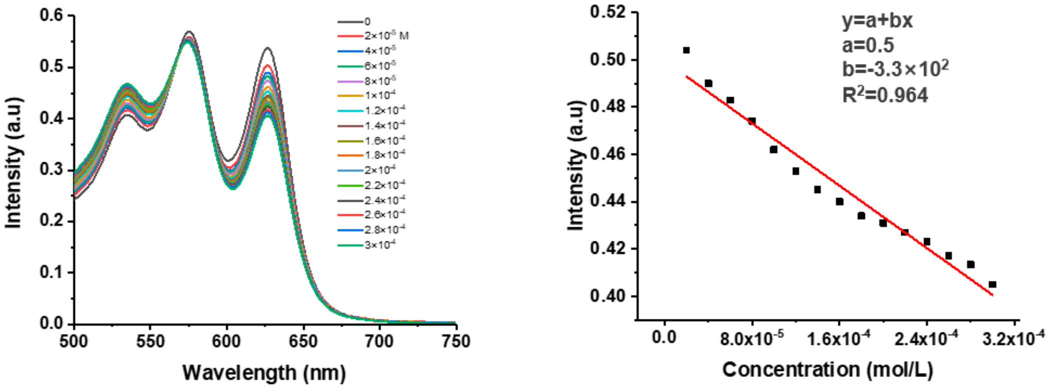

Figure S8. Detection of purine using UV-vis absorption-based sensing assay

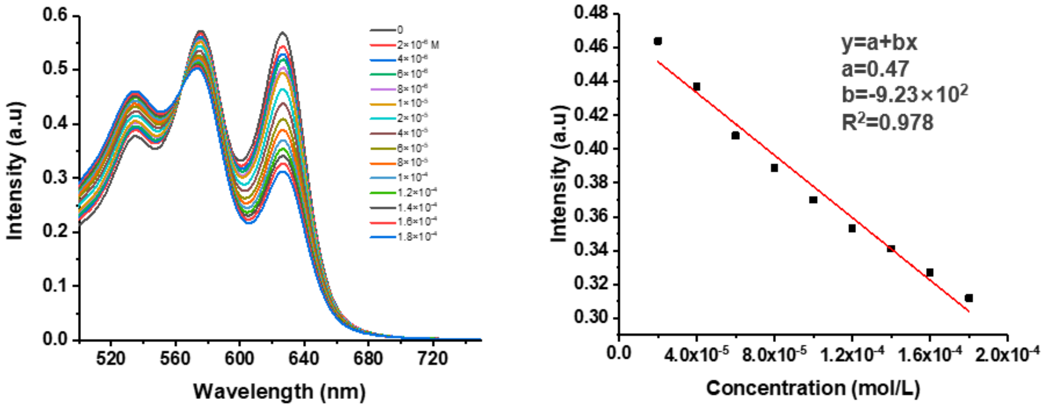

Figure S9. Detection of quinoline using UV-vis absorption-based sensing assay

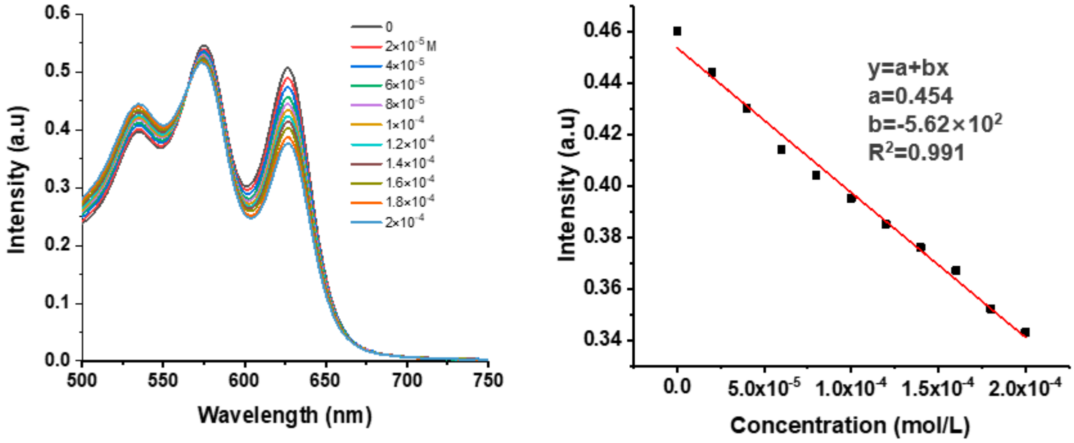

Figure S10. Detection of indole using UV-vis absorption-based sensing assay

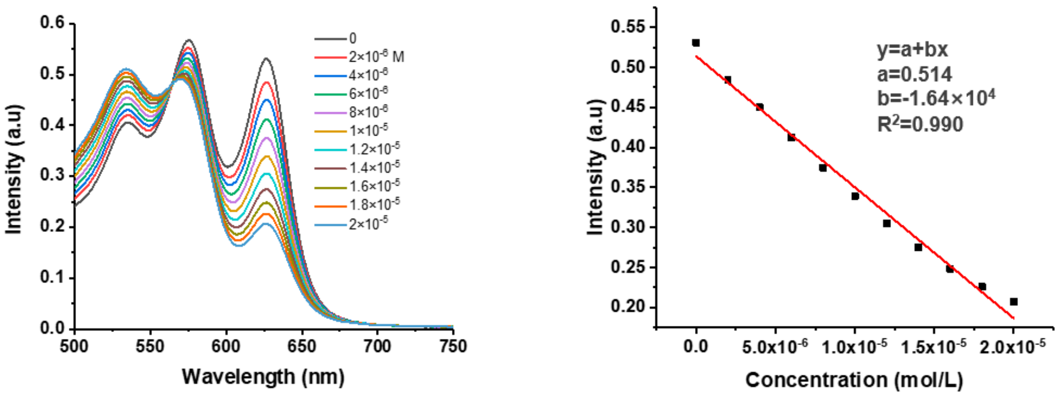

Figure S11. Detection of tetracycline using UV-vis absorption-based sensing assay

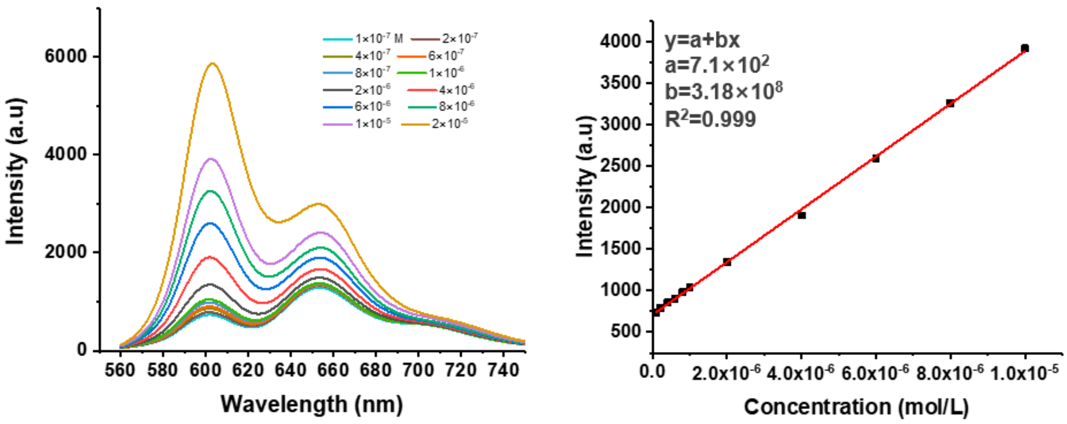

Figure S12. Detection of butylamine using PL-based sensing assay

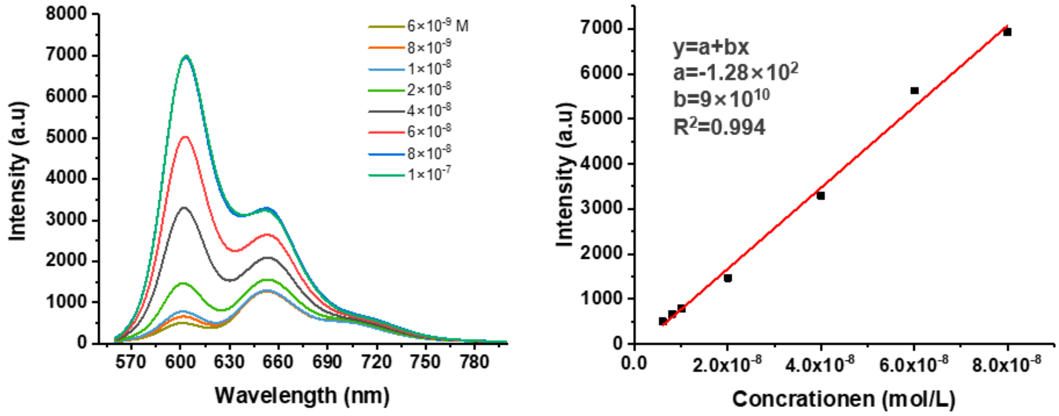

Figure S13. Detection of spermine using PL-based sensing assay

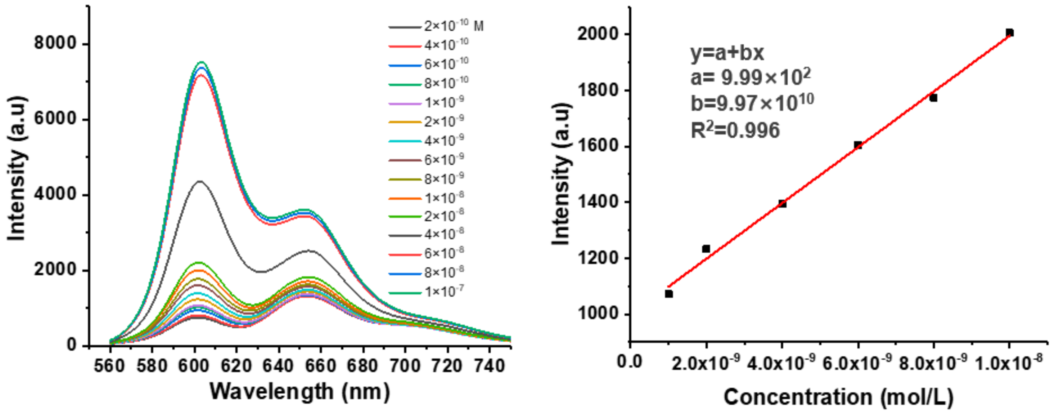

Figure S14. Detection of aniline using PL-based sensing assay

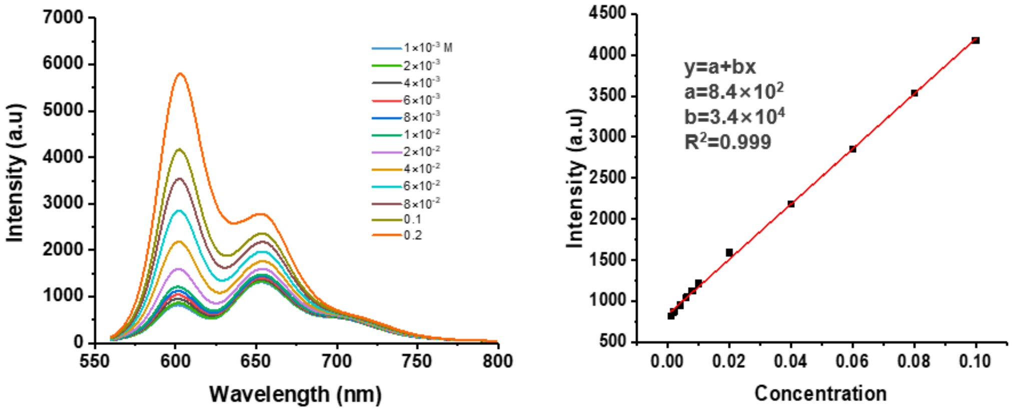

Figure S15. Detection of pyrrole using PL-based sensing assay

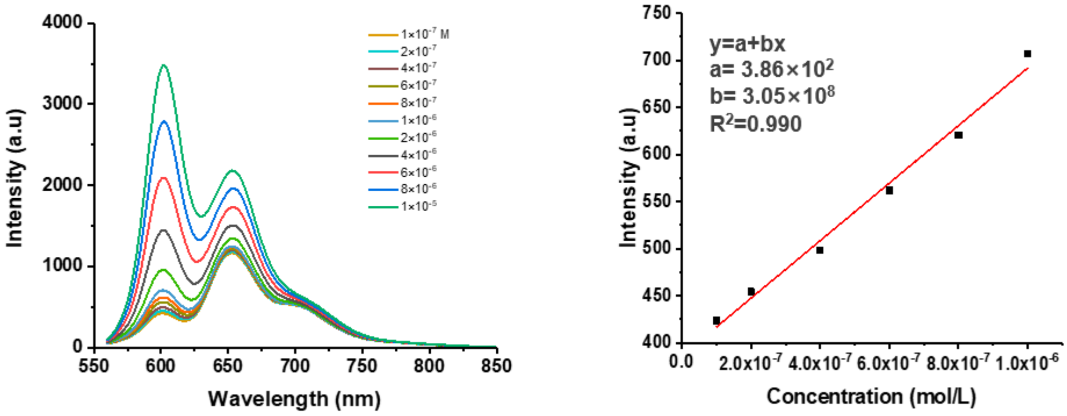

Figure S16. Detection of imidazole using PL-based sensing assay

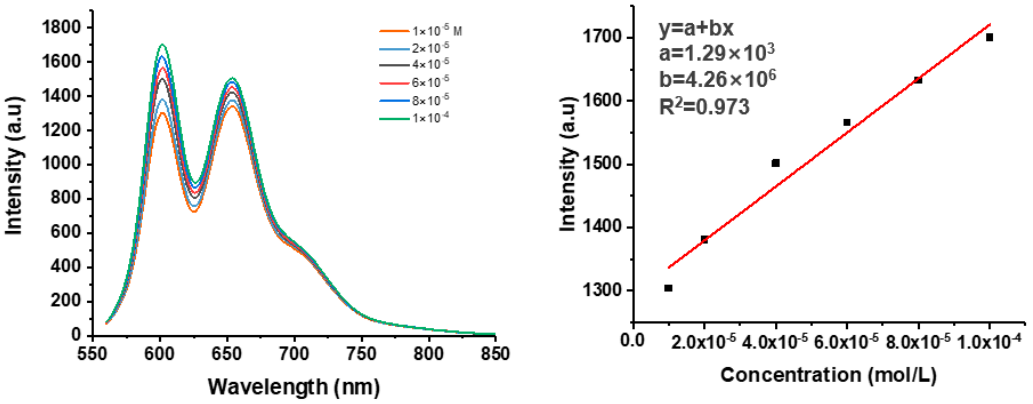

Figure S17. Detection of purine using PL-based sensing assay

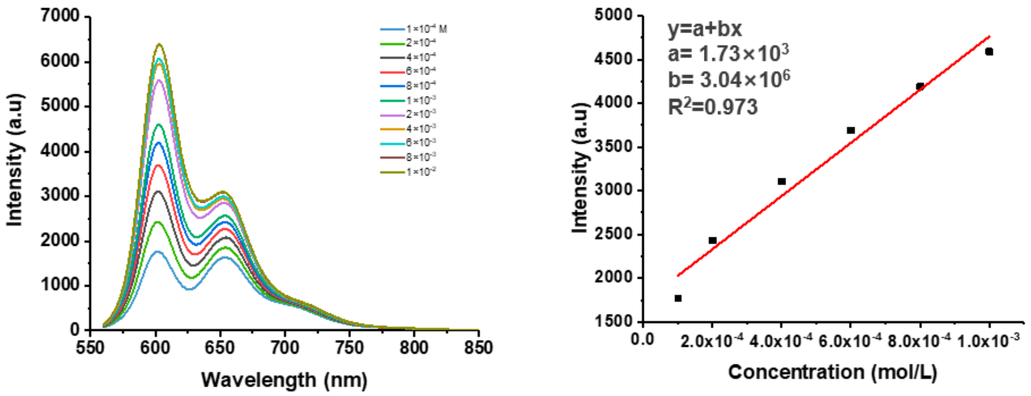

Figure S18. Detection of quinoline using PL-based sensing assay

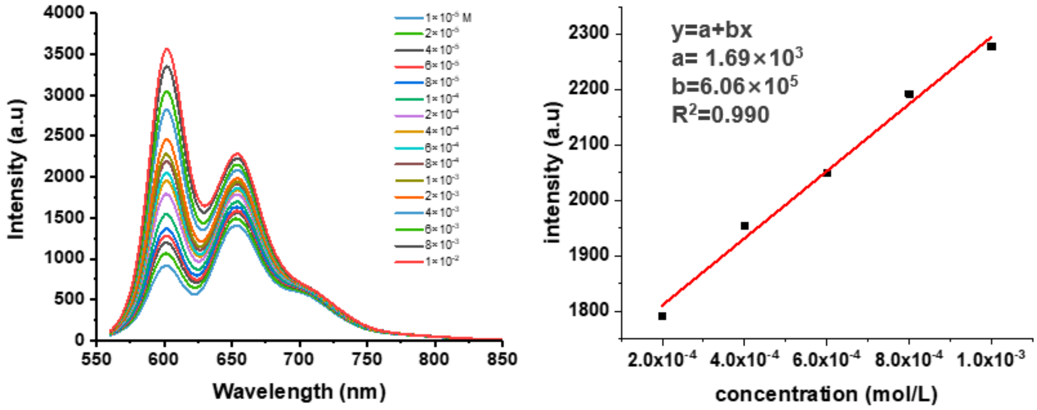

Figure S19. Detection of indole using PL-based sensing assay

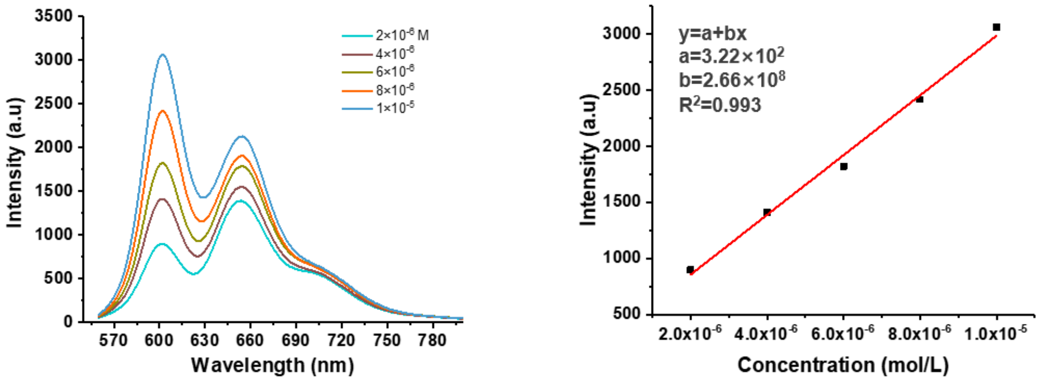

Figure S20. Detection of tetracycline using PL-based sensing assay

Table S1. Summary of differences between calculated values and the actual values in percentage (Diff. %) of UV-vis absorption- and PL-based assays for the detection of various NCOBs

NCOBs[a]	Diff. % (UV-Vis)			Diff. % (PL)		
butylamine	0.4 uM	1.4 uM	2.2 uM	0.15uM	5.0 uM	9 uM
	1.6%	0.6%	0.8%	9.7%	5%	0.4%
spermine	3 nM	9 nM	17 nM	7 nM	25 nM	70 nM
	1.1%	0.7%	0.9%	1.2%	4.4%	1.8%
aniline	3 nM	11 nM	17 nM	1.5 nM	5 nM	9 nM
	3.6%	0.9%	3.8%	8.9%	2.7%	7%
pyrrole	0.3 mM	1.1 mM	1.7 mM	1.5 mM	50 mM	90 mM
	3.7%	0.7%	1.2%	9.7%	3.8%	4.8%
imidazole	0.3 uM	0.9 uM	1.5 uM	0.15 uM	5 uM	9 uM
	1.1%	0.7%	3.4%	4.5%	6.2%	4.7%
pyridine	3 nM	9 nM	17 nM	1.5 nM	25 nM	50 nM
	3.9%	0.26%	2.3%	9.8%	8.3%	2.2%
purine	0.05 mM	0.15 mM	0.27 mM	15 uM	50 uM	90 uM
	5.8%	8.9%	8.3%	9.7%	3.6%	4.7%
quinoline	30 uM	90 uM	170 uM	0.15 mM	0.5 mM	0.9 mM
	8.3%	3.5%	4.5%	7.2%	5.2%	3%
indole	30 uM	90 uM	170 uM	0.3 mM	0.6 mM	0.9 mM
	6.7%	2.8%	5.7%	9.5%	2.3%	2.6%
lysine	1.5 uM	5.5 uM	1.7 uM	0.25 uM	9 uM	15 uM
	0.68%	1.57%	1.99%	9.5%	5.5%	3%
tetracycline	3 uM	11 uM	17 uM	3 uM	6 uM	9 uM
	5.7%	4.1%	1.9%	3.7%	5.5%	3.3%

[a] for each NCOBs, top row presented are the actual concentrations of the NCOBs; and the bottom row presented are the differences in percentage of the calculated values from the calibration curves versus the actual values.

Table S2. Comparison of the performance of NCOBs sensing reported in this work to that of prior art.

	NCOBs	LOD [a] (**this work**)[b]	LOD [a] (**prior art**)[c]	Ref.
1	butylamine	5.50×10^{-2} uM	18 uM	[1]
2	spermine	0.50 nM	1.75 nM	[2]
3	aniline	0.53 nM	6.8 uM	[3]
4	pyrrole	0.12 mM	3.5 uM	[4]
5	imidazole	74 nM	2.65 nM	[5]
6	pyridine	0.68 nM	0.21 uM	[6]
7	purine	12.4 uM	6 uM	[7]
8	quinoline	15 uM	-	-
9	indole	25 uM	-	-
10	lysine	0.20 uM	19 uM	[8]
11	tetracycline	0.20 uM	5.6 nM	[9]

[a]LOD, limit of detection (3σ). [b]the results listed here are the best ones achieved in this work from either UV-Vis- or PL-based sensing. [c]only best performance assays found in literature are listed here, which means the work listed here have the lowest LOD among all the work reported.

References

1. Yang, X.; Wang, Y.; Fu, H.; Wang, W.; Han, D.; An, X. Experimental and theoretical study on the excellent amine-sensing performance of Au decorated WO3 needle-like nanocomposites. *Mater. Chem. Phys.* **2019**, *234*, 122–132.
2. Bhamore, J.R.; Murthy, Z.; Kailasa, S.K. Fluorescence turn-off detection of spermine in biofluids using pepsin mediated synthesis of gold nanoclusters as a probe. *J. Mol. Liq.* **2019**, *280*, 18–24.
3. Feng, H.-J.; Xu, L.; Liu, B.; Jiao, H. Europium metal–organic frameworks as recyclable and selective turn-off fluorescent sensors for aniline detection. *Dalton Trans.* **2016**, *45*, 17392–17400.
4. Park, S.; Lee, S.-Y. Photoluminescent microrods from the self-assembly of a biomimetic molecule: Application for the optical detection of pyrrole. *Sens. Actuators B*. **2014**, *202*, 690–698.
5. Manshaei, F.; Bagheri, H.; Eshaghi, A. Turn-off chelation-enhanced fluorescence sensing of carbon dot-metallic deep eutectic solvent by imidazole-based small molecules. *Sens. Actuators B* **2021**, *344*, 130228.
6. Ahmed, H.M.; Ghali, M.; Zahra, W.K.; Ayad, M. Optical sensing of pyridine based on green synthesis of passivated carbon dots. *Mater. Today Proc.* **2020**, *33*, 1845–1848.
7. Salve, M.; Amreen, K.; Rajurkar, P.; Pattnaik, P. K.; Goel, S. Miniaturized Disposable Buckypaper-Polymer Substrate Based Electrochemical Purine Sensing Platform. *ECS J. Solid State*

Sci. Technol. **2020**, *9*, 101009.

8. Pandey, S.P.; Singh, P.K. A polyelectrolyte based ratiometric optical sensor for arginine and lysine. *Sens. Actuators B* **2020**, *303*, 127182.
9. Velmurugan, S.; Zhi-Xiang, L.; Yang, T. C.; Juan, J. C. Rational design of built-in stannic oxide-copper manganate microrods p-n heterojunction for photoelectrochemical sensing of tetracycline. *Chemosphere* **2021**, *271*, 129788.
